# DECIPHER: Supporting the interpretation and sharing of rare disease phenotype‐linked variant data to advance diagnosis and research

**DOI:** 10.1002/humu.24340

**Published:** 2022-02-21

**Authors:** Julia Foreman, Simon Brent, Daniel Perrett, Andrew P. Bevan, Sarah E. Hunt, Fiona Cunningham, Matthew E. Hurles, Helen V. Firth

**Affiliations:** ^1^ Human Genetics Programme, Wellcome Sanger Institute Wellcome Genome Campus Cambridge UK; ^2^ European Molecular Biology Laboratory, European Bioinformatics Institute Wellcome Genome Campus Cambridge UK; ^3^ Department of Medical Genetics, Cambridge University Hospitals NHS Foundation Trust Cambridge Biomedical Campus Cambridge UK

**Keywords:** genetic disorders, genomic medicine, genotype phenotype correlation, Matchmaker Exchange, rare diseases, variant interpretation, whole‐exome sequencing, whole‐genome sequencing

## Abstract

DECIPHER (https://www.deciphergenomics.org) is a free web platform for sharing anonymized phenotype‐linked variant data from rare disease patients. Its dynamic interpretation interfaces contextualize genomic and phenotypic data to enable more informed variant interpretation, incorporating international standards for variant classification. DECIPHER supports almost all types of germline and mosaic variation in the nuclear and mitochondrial genome: sequence variants, short tandem repeats, copy‐number variants, and large structural variants. Patient phenotypes are deposited using Human Phenotype Ontology (HPO) terms, supplemented by quantitative data, which is aggregated to derive gene‐specific phenotypic summaries. It hosts data from >250 projects from ~40 countries, openly sharing >40,000 patient records containing >51,000 variants and >172,000 phenotype terms. The rich phenotype‐linked variant data in DECIPHER drives rare disease research and diagnosis by enabling patient matching within DECIPHER and with other resources, and has been cited in >2,600 publications. In this study, we describe the types of data deposited to DECIPHER, the variant interpretation tools, and patient matching interfaces which make DECIPHER an invaluable rare disease resource.

## Graphical abstract

The DECIPHER web platform supports the sharing and interpretation of rare disease phenotype‐linked variant data to advance diagnosis and research.

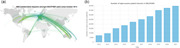

## 1 INTRODUCTION

The population prevalence of rare diseases has recently been estimated to be 3.5%–5.9%, which equates to 263–446 million people affected globally. A large proportion of these rare diseases, approximately 72%, are known to have a genetic basis (Nguengang Wakap et al., 2020). Advances in genomic technologies to determine causal variants, such as whole‐exome sequencing, currently identify the genetic basis of disease for only 25%–47% of patients (Liu et al., 2019; Quaio et al., 2020; Sawyer et al., 2016; Stranneheim et al., 2021). As a result, many patients undergoing diagnostic genetic testing do not receive a molecular diagnosis, and often experience long delays which have a substantial emotional impact on the family (Miller, 2021) and significant healthcare costs (Monroe et al., 2016). A molecular diagnosis has multiple benefits for the patient and their family, including better understanding of the prognosis, personalized treatment, tailored management and surveillance, improved access to health and social care, and increased reproductive choice (Liu et al., 2019; Wright et al., 2018).

The number of rare Mendelian diseases with known molecular etiology is estimated to be 5000–6000 (Hartley et al., 2018); however, for the majority of disease‐associated genes, it is not known which variants are disease‐causing, and which are benign. Different pathogenic variants in the same gene can cause different diseases, for example, variants in *FGFR3* can cause multiple diseases including Muenke Syndrome, Hypochondroplasia, Achondroplasia, Camptodactyly Tall Stature and Hearing Loss syndrome (CATSHLS), Lacrimo‐Auriculo‐Dento‐Digital Syndrome (LADD Syndrome), Thanatophoric Dysplasia (types 1 & 2), SADDAN syndrome and Crouzon Syndrome with Acanthosis Nigricans. Different diseases caused by variants in the same gene must be considered distinct due to their disparate clinical presentation and different treatment options. The sharing of patient‐level variants and phenotypes is therefore essential to accelerate our understanding of the molecular basis of genetic disease.

**Figure 1:**
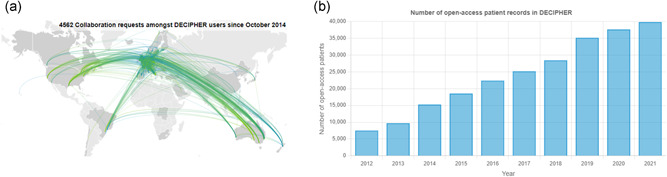
(a) The DECIPHER community is a global network of academic clinical centers with expertise in genetics. Depositing centers are able to send messages directly to other registered users about patient matches through DECIPHER. Since October 2014 over 4500 such messages have been sent. Here, each line represents a collaboration request sent between depositing centers. Unregistered users’ messages, sent through DECIPHER, are not included in this image. (B) The DECIPHER database currently openly shares approx. 40,000 rare disease patient records, built up over time

DECIPHER (Bragin et al., 2014; Chatzimichali et al., 2015; Firth et al., 2009; Swaminathan et al., 2012) is a global web‐based platform that shares phenotype‐linked variant data from rare disease patients (Figure 1a). It is freely available via a web interface at https://www.deciphergenomics.org. Approximately 40,000 of the patient records held by DECIPHER have explicit patient consent for open sharing on the website (Figure 1b). These openly shared records contain more than 51,000 variants and more than 172,000 phenotype terms. The integration of this phenotype and variant data enables the discovery of new gene‐disease trait and variant‐disease trait relationships, driving molecular diagnosis and our understanding of human biology. Since DECIPHER was established in 2004, the platform has been used and cited in more than 2,600 published manuscripts.

Patient records in DECIPHER are deposited by academic clinical centers, which are affiliated both to a hospital that oversees the treatment of patients with genetic conditions, and to a local university department of human/clinical genetics. Eligible centers can apply to join DECIPHER using an online application form (https://www.deciphergenomics.org/join/overview). Data from a center is stored within a DECIPHER project, and a senior clinician at that center (clinical coordinator), sometimes in conjunction with a senior clinical scientist (lab coordinator), has the responsibility for approving/rejecting applications from individuals working at that center who wish to access the data in the project.

The platform supports the deposition of genetic and genomic variation (e.g., sequence variants, insertions and deletions, short tandem repeats (STRs), copy‐number variants [CNVs], complex and copy number neutral structural variants); including that observed in genomic conditions. Variant interpretation interfaces are provided, including genome and protein browsers, which contextualize genetic and phenotype information to enable accurate interpretation. These interfaces integrate external data sets such as the Genome Aggregation Database (gnomAD; Karczewski et al., 2020), which can be used to exclude variants seen at appreciable frequency in the general population, in addition to disease relevant data sets such as ClinVar (Landrum et al., 2018) and DECIPHER records themselves. DECIPHER also encourages the use of global standards to promote good practice, including the American College of Medical Genetics and Genomics and Association of Molecular Pathology (ACMG/AMP) guidelines for sequence variant interpretation (Richards et al., 2015) and ACMG/ClinGen technical standards for interpreting CNVs (Riggs et al., 2020).

In the following sections, we present examples of the genotype/phenotype data deposited and shared with the rare disease community. In addition, we present the tools provided by DECIPHER to assess the pathogenicity of variants according to international standards, and the utility of DECIPHER to map the clinically relevant part of the assayable human genome.

## 2 DECIPHER PATIENT RECORDS

DECIPHER associates variants and phenotypes through individual patient records, each of which are connected to a particular depositing center. DECIPHER itself cannot reidentify individuals, and technical and organizational measures are in place to safeguard data. These measures are reviewed and updated in line with evolving best practices.

On deposition, each patient record is given a DECIPHER Patient ID as a reference, which is shown on the website and forms part of the URL for the patient record (e.g., https://www.deciphergenomics.org/patient/283351—note that URLs of the form https://decipher.sanger.ac.uk/patient/283351 continue to be supported). Each patient record also has an internal ID (e.g., a lab number), which is only displayed to users of the depositing center. The internal ID allows the depositing center (only) to link the record to an individual patient.

Through the DECIPHER platform, it is possible to send a patient's clinician an email to request further information about the patient, for example in the case where there is a potential patient match, or if a researcher is carrying out a functional study on the gene in which that patient's variant is situated. Below we will describe in more detail the clinical and research utility of this notification system.

## 3 DEPOSITION AND BREADTH OF SHARING

DECIPHER has been carefully designed to ensure that the depth and breadth of sharing are proportionate to the scientific/clinical needs and level of consent. For example, a user who does not belong to a DECIPHER project can only access the openly shared patient data, while data that is visible to registered users who are logged in reflects their project and consortium memberships.

Patient genotype and phenotype data can be deposited to DECIPHER in three ways:

1. Via the web interface for an individual patient's data.
2. By uploading Excel or csv files via the web interface (bulk upload) for data from multiple patients.
3. Using the deposition API to allow programmatic uploading of data and synchronization of data across systems (e.g., synchronization between a center's electronic health records and the patient records in that center's DECIPHER project).

DECIPHER users at the depositing center determine the sharing level of each patient record and variant. Patient records, and individual variants within these records, can be kept private to the depositing center. This allows DECIPHER's tools to be used for assessing variant pathogenicity to inform the conversation with the patient before seeking consent for wider sharing. With explicit patient consent, patient records are shared openly, with the data available to anyone who visits the website. Consent forms approved by the English National Research Ethics Service (NRES) are available to download from the DECIPHER website. Since DECIPHER is an international database, depositing centers must ensure appropriate consent is obtained in accordance with local laws and regulations. DECIPHER also supports consortium sharing. This allows sharing of patient records between a defined group of centers, where there is an expectation of collaboration for patient care, again before explicit patient consent for open sharing has been obtained. DECIPHER currently hosts six consortia, which share more than 63,000 patient records. Consortia include the United Kingdom National Health Service consortium, the Deciphering Developmental Disorders (DDD) consortium which shares research data from the DDD study (Wright et al., 2015), and a data‐sharing consortium covering New South Wales and Western Australia.

DECIPHER is a live interface and data deposited is available to view, interpret, and share in real time. Patient records can be added and edited iteratively as more information becomes available, for example, additional phenotype terms, the inheritance status of a variant, or new functional data. Depositors are encouraged to ensure complete and accurate data entry, for the benefit of all users of DECIPHER. If a patient is reported in a publication, submitters are requested to add the citation to the patient record to alleviate issues of double‐counting of cases. Information can be added to a record by a clinician and clinical scientist working asynchronously and in different locations.

## 4 GENETIC DATA

**Figure 2:**
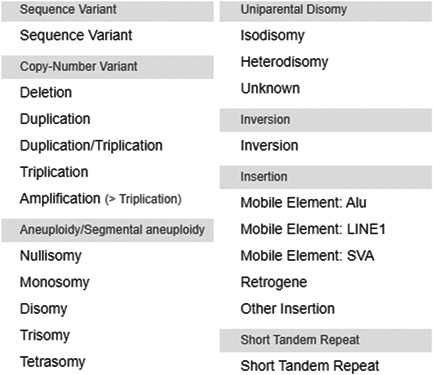
DECIPHER supports the deposition and sharing of almost all types of genetic variation

As our knowledge of rare disease genetics develops and the interaction between gene loci is more fully understood, there is a pressing need for the visualization of all types of genetic variation within a single interface. DECIPHER fulfills this need, supporting many types of genetic variation including sequence variants, CNVs, aneuploidy, uniparental disomy (UPD), inversions, insertions, and STRs (Figure 2). The visualization of Complex Genomic Rearrangements is challenging and thus not every genetic rearrangement can yet be supported.

### 4.1 Variant deposition

Variants are deposited using genomic coordinates. Sequence variants can also be deposited using a relevant subset of HGVS nomenclature (den Dunnen et al., 2016), and will be normalized (left aligned, parsimonious) during the deposition process (Tan et al., 2015). For known STRs, the disease‐relevant STR can be selected from a dropdown in the web interface. Additional information about the variant such as inheritance, genotype, pathogenicity, and contribution to phenotype can also be recorded.

### 4.2 Mosaicism

For de novo mosaic variants, it is possible to record the mosaicism observed in each tissue, as a percentage. This information is clinically important as it can help explain the variability of clinical symptoms, for example, the difference between nevus sebaceous or Schimmelpenning syndrome (where extracutaneous abnormalities are present), caused by *HRAS* and *KRAS* variants (Groesser et al., 2012).

### 4.3 Mitochondrial variants

DECIPHER supports the deposition and interpretation of variants in the nuclear and mitochondrial genomes. Mitochondrial diseases are the most common form of inherited neuro‐metabolic disorders and are caused by mutations in the nuclear or mitochondrial genomes. In addition, nuclear genetic factors have been shown to influence clinical outcomes for mitochondrial DNA mutations (Boggan et al., 2019). Thus the display of both genomes in a single interface is clinically important. In DECIPHER it is possible to record homoplasmy or the percentage of heteroplasmy per tissue, which is clinically essential as it has been shown to contribute to disease progression (Grady et al., 2018).

### 4.4 Variant haplotypes

Variants may work *in cis* to create or modify a disease allele or *in trans* to cause a biallelic disorder. For this reason, DECIPHER users can assign variants to a haplotype, for example, for compound heterozygous variants, the variants will be shown as *in trans*. As our understanding of rare disease genetics improves, the representation of its complexity is becoming even more essential. It is known that genetic modifiers alleviate or exacerbate the severity of the disease (Rahit & Tarailo‐Graovac, 2020) and there are recent examples where rare pathogenic haplotypes have been shown to cause disease, such as an albinism‐causing TYR haplotype (Campbell et al., 2019).

### 4.5 Pathogenicity predictors

For all sequence variants deposited to DECIPHER, predictions from the Ensembl Variant Effect Predictor (VEP; McLaren et al., 2016) are displayed across all Ensembl/GENCODE transcripts. Predictions include the consequence (e.g., missense, frameshift), the protein change, and several pathogenicity scores: SIFT (Sim et al., 2012), PolyPhen‐2 (Adzhubei et al., 2013), CADD (Kircher et al., 2014), REVEL (Ioannidis et al., 2016), and SpliceAI (Jaganathan et al., 2019). DECIPHER seeks advice from experts in the field and refers to benchmarking studies for pathogenicity predictors (e.g., Gunning et al., 2021) before the inclusion of additional scores, assisting in the application of good practice.

### 4.6 Reference genome

All genomic information is displayed in the GRCh38 assembly version of the human genome, allowing the most up‐to‐date genome and transcript information to be used to enable accurate variant interpretation. The display of genomic data in GRCh38 permits DECIPHER to promote the use of Matched Annotation from NCBI and EMBL‐EBI (MANE) transcripts, where the RefSeq and Ensembl/GENCODE transcripts from a protein‐coding gene pair are identical (5′ UTR, coding region, and 3′ UTR). DECIPHER currently promotes and highlights MANE Select transcripts, one high‐quality representative transcript per protein‐coding gene that is well‐supported by experimental data and represents the biology of the gene (https://tark.ensembl.org/web/mane_project). Describing variants relative to a single, recommended transcript, along with sequence variant normalization, assists in the standardization of variant reporting.

### 4.7 Reference conversion tools

**Figure 3:**
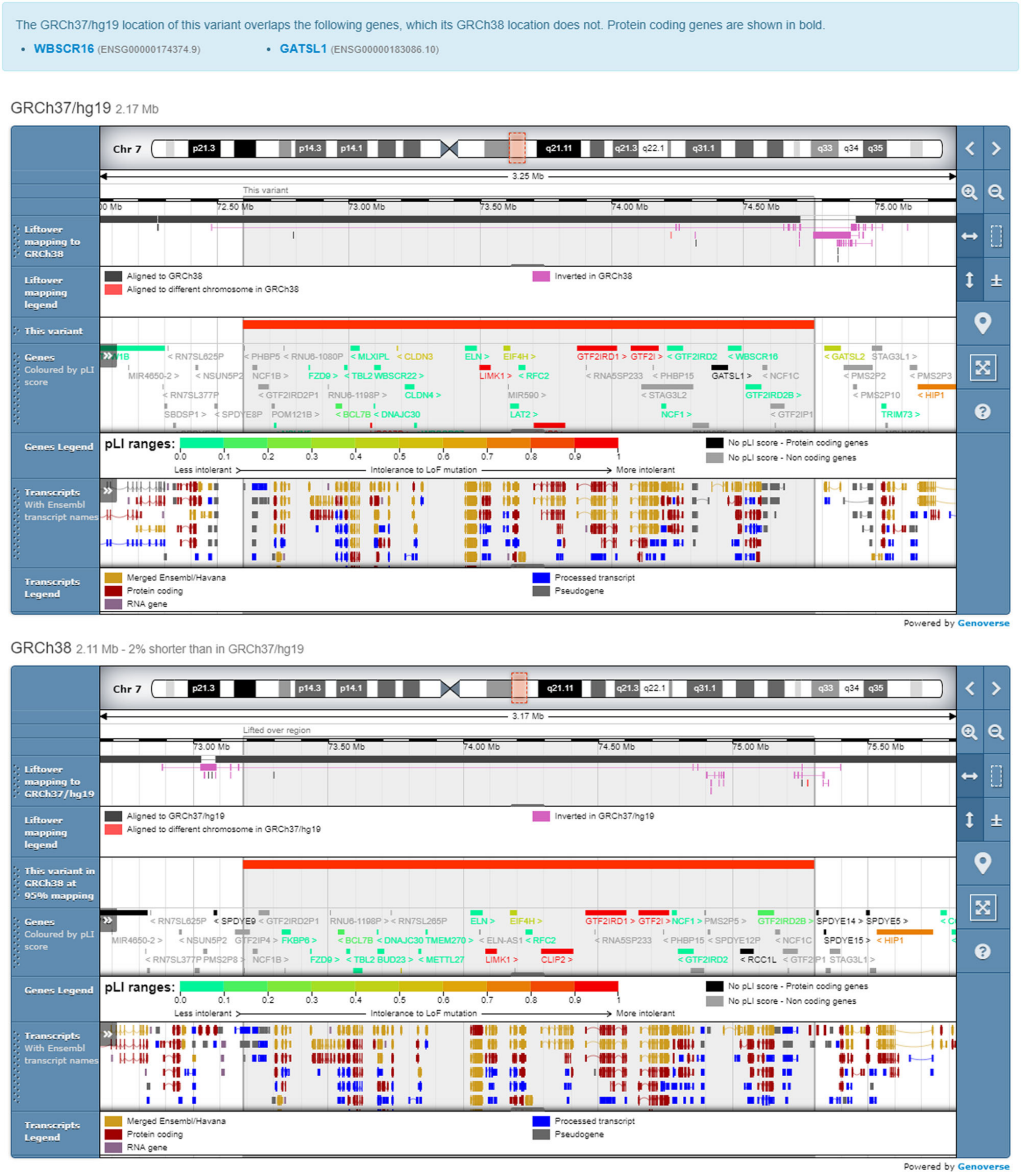
All genomic data is visualized in GRCh38, but deposition is still supported in GRCh37/hg19. Tools are provided to visualize the differences between assemblies. These include comparative genome browsers and gene lists for variants lifted over by DECIPHER, and a liftover mapping genome browser track

Deposition with GRCh37/hg19 coordinates is still supported: before normalization, DECIPHER remaps GRCh37 coordinates onto the GRCh38 assembly, using an algorithm based on the UCSC LiftOver tool (https://genome.ucsc.edu/cgi-bin/hgLiftOver; Kuhn et al., 2013). A recent study comparing exome variant calls detected in GRCh37 and GRCh38 genome assemblies, with lifted over variants (GRCh37 to GRCh38), has shown that the majority of variants have concordant genotypes (>98% SNVs and >93% indels across all samples), with most discordant calls clustered within discrete discordant reference patches (Li et al., 2021). DECIPHER provides a range of tools to allow users to visualize the differences between assemblies and help identify regions of discordance between the assemblies. These include GRCh37 and GRCh38 comparative genome browsers, gene lists for variants lifted over by DECIPHER which display genes that no longer overlap the variant, and a liftover mapping genome browser track (Figure 3).

## 5 PHENOTYPIC DATA

**Figure 4:**
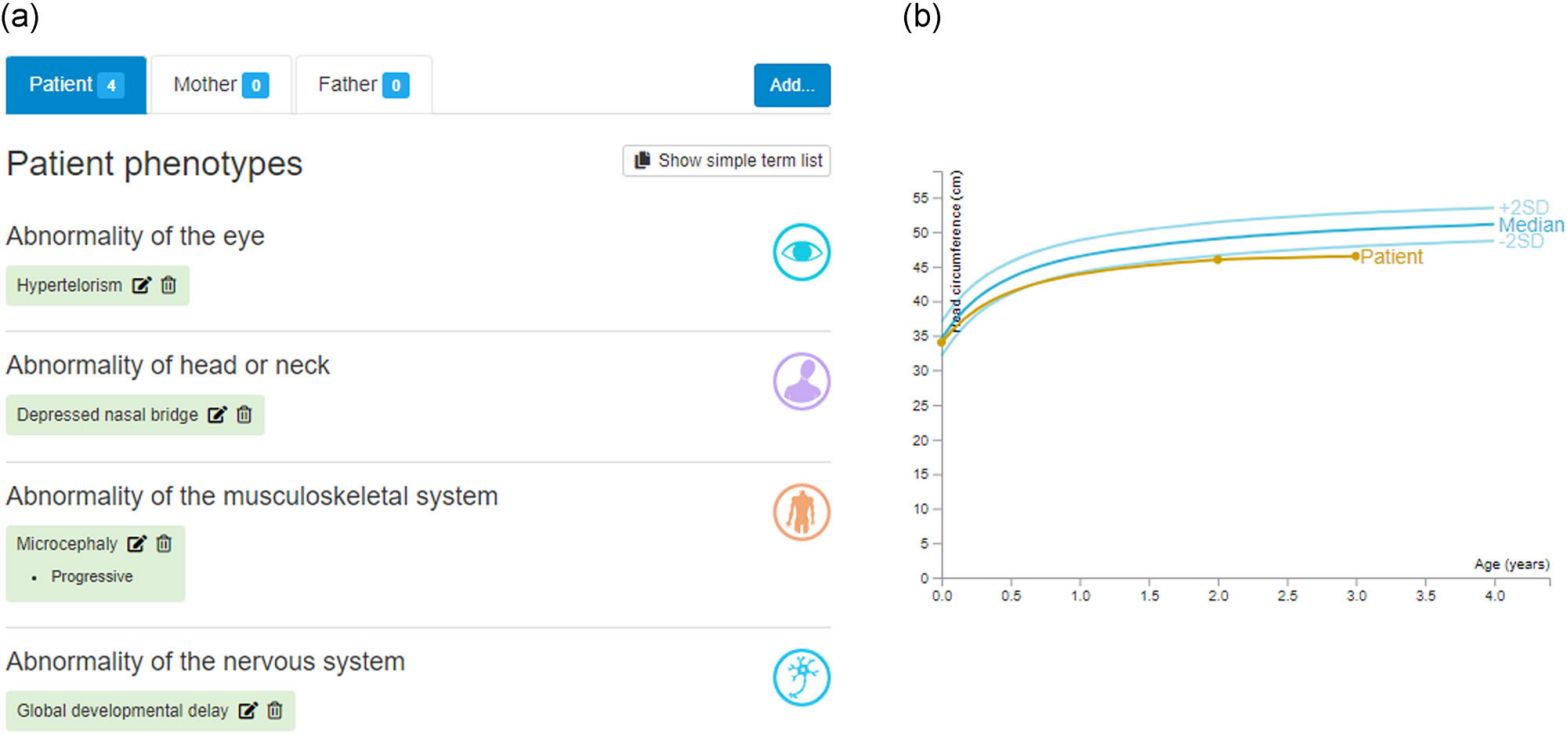
(A) DECIPHER enables the deposition of phenotypes using HPO terms. (B) DECIPHER supports the deposition of developmental milestones and anthropometric measurements, for example, occipitofrontal (head) circumference

DECIPHER supports detailed phenotype data capture (Figure 4a) which enables the in‐depth comparison of patient phenotypes, as well as the delineation of new syndromes. Much of the phenotype is represented using Human Phenotype Ontology (HPO) terms—a standardized, controlled vocabulary that supports deep phenotyping (Köhler et al., 2019). This allows phenotypic information to be described unambiguously, and for phenotypic similarity between patients to be established computationally by comparing related terms in the ontology. This is essential for finding potential patient matches. The DECIPHER phenotype deposition interface provides a search tool, allowing HPO terms to be added to a patient record quickly and easily. DECIPHER also supports the recording of the absence of clinically relevant phenotypes, and of manifestations of HPO terms (clinical modifiers), such as severity, age of onset, and pace of progression. This information can be helpful to users trying to determine the accuracy of a patient match, especially when the number of patient phenotypes is small.

In collaboration with ophthalmologists, DECIPHER has developed forms for groups of HPO phenotypes for the eye community, to assist phenotyping in the clinic. These forms contain a predetermined list of HPO terms that can be marked absent or present, and include common retinal and non‐retinal disease, and symptoms and signs (extraocular features, ocular features, and electrodiagnostic testing and imaging). These forms are available to depositors as an optional addition to the phenotyping interface. Clinical data from >1500 individuals with inherited eye disorders have been deposited to DECIPHER using the relevant phenotype form. DECIPHER is working with other disease specialties to develop further forms.

### 5.1 Family history

In the case of inherited disorders, it is important to capture family phenotype history. In DECIPHER, users can record whether or not relevant family members are affected with similar or related phenotypes. Presence of absence of HPO terms can also be indicated for each family member if known.

### 5.2 Quantitative data

In addition to HPO terms, DECIPHER supports quantitative phenotype data capture (Figure 4b). Developmental milestones (age of social smile, sat independently, walked independently, and first words) and anthropometric measurements (growth, visual function, fundus imaging) can be deposited. Aggregated observations from open‐access patient records are shared openly (see Section 7.3). DECIPHER also provides an interface to record birth and pregnancy information, such as age of the mother/father at birth of the patient, consanguinity, maternal illness, and gestation (which is also used to adjust growth charts); this information is not currently shared openly, but is shared within a consortium.

## 6 GENOTYPIC SUMMARIES TO ASSIST VARIANT INTERPRETATION

DECIPHER provides a suite of tools to assist in assessing the pathogenicity of variants, including genome and protein browsers.

### 6.1 Protein browser

**Figure 5:**
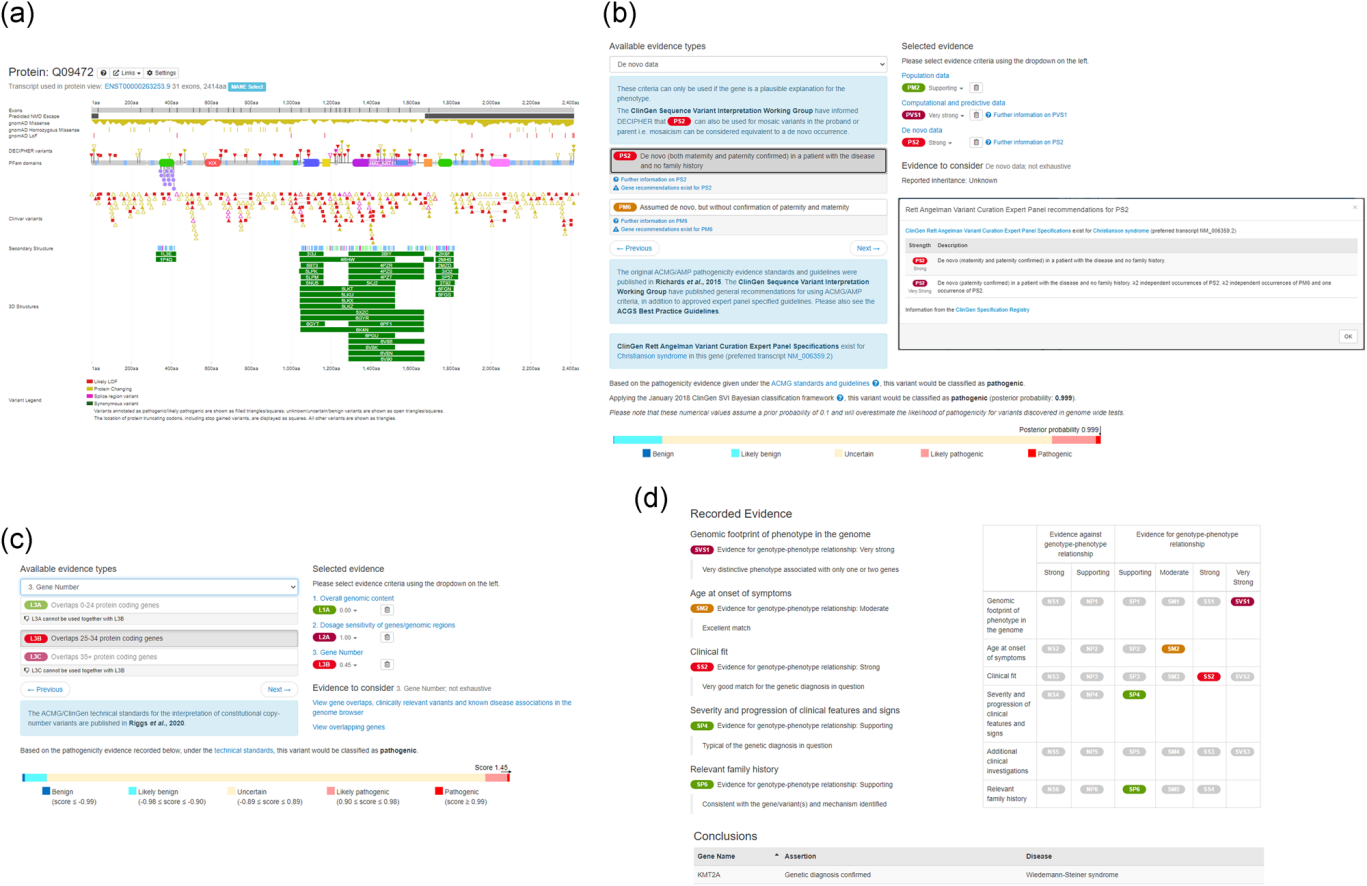
(A) DECIPHER has developed a protein browser that summarizes genotypic data. Tracks include: Pfam domains, DECIPHER and ClinVar variants, gnomAD variants, and region of predicted nonsense‐mediated decay (NMD) escape. (B) DECIPHER supports the annotation and sharing of sequence variant pathogenicity assessments using ACMG guidelines. A pathogenicity evidence interface is available for depositors. Relevant criteria are selected by clicking on the criteria displayed on the left under “Available evidence types.” “Selected criteria” are displayed on the right, along with “Evidence to consider.” “Further information” links provide recommendations for the use of criteria. In this example, a variant in SLC9A6 is being annotated and ClinGen Variant Curation Expert Panel specifications exist for this gene. Detailed information about these recommendations are displayed by clicking on the “Gene recommendation” links—expert panel recommendations for de novo criterion PS2 are displayed. As criteria are added, DECIPHER calculates the variant pathogenicity according to criteria‐combining rules detailed in the original 2015 guidelines, and according to the ClinGen SVI Working Group's Bayesian classification framework. (C) DECIPHER supports the annotation of copy‐number variants according to ACMG/ClinGen technical standards. Similar to the sequence variant interface, “Available evidence types” are displayed on the left, with “Selected evidence” and “Evidence to consider” displayed on the right. As criteria are selected, the classification score and pathogenicity are calculated and displayed at the bottom of the interface. (D) An assessment interface is provided which is designed to be used in a multidisciplinary team meeting to evaluate whether one or more variants explain the clinical features seen in a patient, and record if a diagnosis has been made (or excluded). Depositors can report several lines of evidence, to weigh evidence for or against a genotype‐phenotype relationship. An OMIM gene‐disease pair and assertion is recorded

A protein browser is available for protein‐coding genes, showing a genotypic summary that helps users to determine if a variant is located in a mutational hot spot or established functional domain (Figure 5a). The protein browser is fully interactive and is customizable via a settings menu. In the center of the protein browser, Pfam domains (Mistry et al., 2021) are displayed allowing users to identify distinct functional/structural elements of the protein. Clinically relevant variants from DECIPHER and ClinVar are plotted above and below the Pfam domains, with annotated pathogenicity and predicted molecular consequence (e.g., missense, likely loss‐of‐function [LOF]) indicated through coloring. In addition to the location of the variants being shown, for likely LOF variants, the location of the protein‐truncating codon is indicated, since this information is essential in determining if a transcript is likely to escape nonsense‐mediated decay (NMD). A predicted (NMD) track is also displayed. The location of variation in the general population is shown through display of gnomAD missense and LOF tracks. Regional missense constraint data are also available (regional missense constraint improves variant deleteriousness prediction, Samocha et al., https://www.biorxiv.org/content/10.1101/148353v1), in addition to exon structure. Protein secondary structures (e.g., locations of helices and turns) and the locations of 3D structures (experimental structures were available from the Protein Data Bank in Europe [PDBe] and predicted structures from Alphafold [Jumper et al., 2021]) are displayed at the bottom of the protein browser. Clicking on these 3D structures will display an interactive 3D protein viewer (Marco Biasini, 2015, pv v1.8.1. Zenodo. 10.5281/zenodo.20980) which provides zooming, panning, and rotation, and hovering over an amino acid with a pointing device identifies the visualized amino acid and position (similar behavior exists for ligands). DECIPHER variants are shown in this 3D view, allowing users to determine, for example, if the variants are all within a DNA binding pocket or enzyme active site.

When looking at the protein browser from a patient record with a sequence variant, the location of the patient's variant is displayed by a vertical line, allowing easy orientation. In the case of a patient with a CNV, the protein browser is accessible from the CNV's genes tab, which displays a table of genes that overlap the CNV, along with other relevant information such as gene/disease association information and predictive scores. Clicking on a row displays further information about that gene, including the protein browser. An additional track is shown on the protein browser, indicating which part of the protein overlaps the CNV.

### 6.2 Genome browser

The Genoverse genome browser (http://genoverse.org), developed by the DECIPHER team, is a portable, interactive, customizable genome browser that allows the user to explore data. It displays a number of tracks containing information relevant to variant pathogenicity assessment such as genes associated with disease phenotypes (as curated and maintained by Online Mendelian Inheritance in Man (OMIM, https://omim.org; Amberger et al., 2019), protein ortholog sequences from Ensembl indicating conservation, transcripts (as maintained by Ensembl), and regional missense constraint. Information from population resources such as gnomAD and Database of Genomic Variants (DGV) Gold (Church et al., 2010) are displayed to enable users to determine if their patient's variant has been observed in healthy individuals. Disease relevant variant tracks are also available, which include DECIPHER sequence variants and CNVs, ClinVar sequence and structural variants, and variants from Human Gene Mutation Database (HGMD) public (Stenson et al., 2020). The tracks which are displayed by default are tailored according to the type of variant being assessed.

## 7 TOOLS SUPPORTING MOLECULAR DIAGNOSTIC ASSESSMENT

### 7.1 Assessing pathogenicity according to international standards

DECIPHER supports the annotation and sharing of variant pathogenicity using ACMG guidelines for sequence variants and ACMG/ClinGen technical standards for CNVs, which helps to standardize the classification of variants across centers. When interpreting a CNV it is possible for users to choose to assess the variant using sequence variant guidelines, which may be more applicable for small CNVs since the distinction between a sequence variant and a CNV is blurred (Brandt et al., 2020).

#### 7.1.1 Criteria selection

In both pathogenicity interfaces (Figure 5a,b), types of evidence (such as population data and functional data) are displayed, along with the relevant evidence criteria used to determine if data supports the variant being pathogenic or benign. Relevant criteria can be selected with a single click. Some of the criteria have additional information links. These either provide information about how the criteria can be used according to the original study (e.g., de novo CNV evidence), or in the case of sequence variants they provide information about ClinGen Sequence Variant Interpretation (SVI) Working Group guidelines (e.g., recommendation for functional assays (PS3/BS3); Brnich et al., 2019). As new guidelines become available these pathogenicity interfaces are updated to provide the latest relevant recommendations. The strength of each criterion can be modified as required in the interface.

#### 7.1.2 Relevant evidence

Within the interfaces, there is a customized section displaying “evidence to consider” which provides information relating to the specific evidence type being assessed. For example, for computational and predictive data evidence, predictive pathogenicity scores (SIFT, PolyPhen‐2, CADD, REVEL, and SpliceAI) are displayed. Links are also provided to relevant DECIPHER interpretation interfaces, for example to the in‐built tolerated population variation calculator, which can be used to determine if a variant observed in the reference sample is too common to cause a given rare variant Mendelian disease trait (Whiffin et al., 2017). External links (e.g., PubMed literature search) are also provided.

#### 7.1.3 Calculation of variant pathogenicity

As criteria are added, DECIPHER uses these to calculate the variant pathogenicity. For sequence variants, this is calculated according to the combining rules detailed in the original 2015 ACMG guidelines. In addition, DECIPHER calculates the posterior probability of pathogenicity and classification according to the ClinGen SVI Working Group's Bayesian classification framework, which provides a mathematical foundation for the combining rules (Tavtigian et al., 2018). DECIPHER highlights cases where these classifications disagree, and ultimately all pathogenicity assessments are made by depositors using their professional discretion. For CNVs, the evidence can be scored according to ACMG/ClinGen technical standards instead.

#### 7.1.4 ClinGen Expert Panel specifications

For some genes, there are ClinGen Variant Curation Expert Panel specifications, which recommend adaptations of the sequence variant ACMG guidelines (e.g., Rett and Angelman‐like Disorders Variant Curation Expert Panel for *MECP2, CDKL5, FOXG1, UBE3A, SLC9A6*, and *TCF4*). When interpreting variants in genes for which these recommendations exist, detailed information about how to apply the criteria is provided along with a link to the relevant Clinical Domain Working Group, so that patients with variants in these genes benefit from interpretation in accordance with these recommended standards.

### 7.2 Confirming variant‐phenotype association and making a molecular diagnosis

DECIPHER provides an assessment interface (Figure 5d) which is designed to be used in a multidisciplinary team meeting to evaluate whether one or more variants explain the clinical features seen in a patient, and record if a molecular diagnosis has been made (or excluded). Depositors can report evidence from several evidence lines, such as the age at presentation or additional clinical investigation, to weigh evidence for or against a genotype‐phenotype relationship. An OMIM gene‐disease pair and assertion is recorded, for example, “genetic diagnosis confirmed,” “uncertain genetic diagnosis,” or “non‐penetrant (or presymptomatic) for a dominant genetic disorder.” The output of the assessment is a date‐stamped report providing the patient's variants and phenotypes, in addition to the diagnosis and evidence on which that diagnosis was made.

There are many published examples of patients having blended phenotypes due to pathogenic variants in more than one gene, for example, in Ferrer et al. (2019), the patient had three independent rare disease diagnoses due to pathogenic variants in *SIN3A* (Witteveen–Kolk syndrome), *FLG* (dermatitis), and *EDAR* (ectodermal dysplasia). A recent study has suggested that multiple molecular diagnoses occur in approximately 5% of cases in which a molecular diagnosis is elucidated (Posey et al., 2017). Blended phenotypes among patients with dual diagnoses include cases where individual phenotypic features are clearly attributable to only one of the two diagnoses, and cases where phenotypic features could be attributable to both of the diagnoses. The assessment interface allows multiple assessments to be created for a patient, allowing the genetic basis of blended phenotypes to be recorded and shared.

### 7.3 Quantitative phenotypic data to confirm fit with diagnosis

#### 7.3.1 Quantitative phenotype data and gene‐specific centile charts

**Figure 6:**
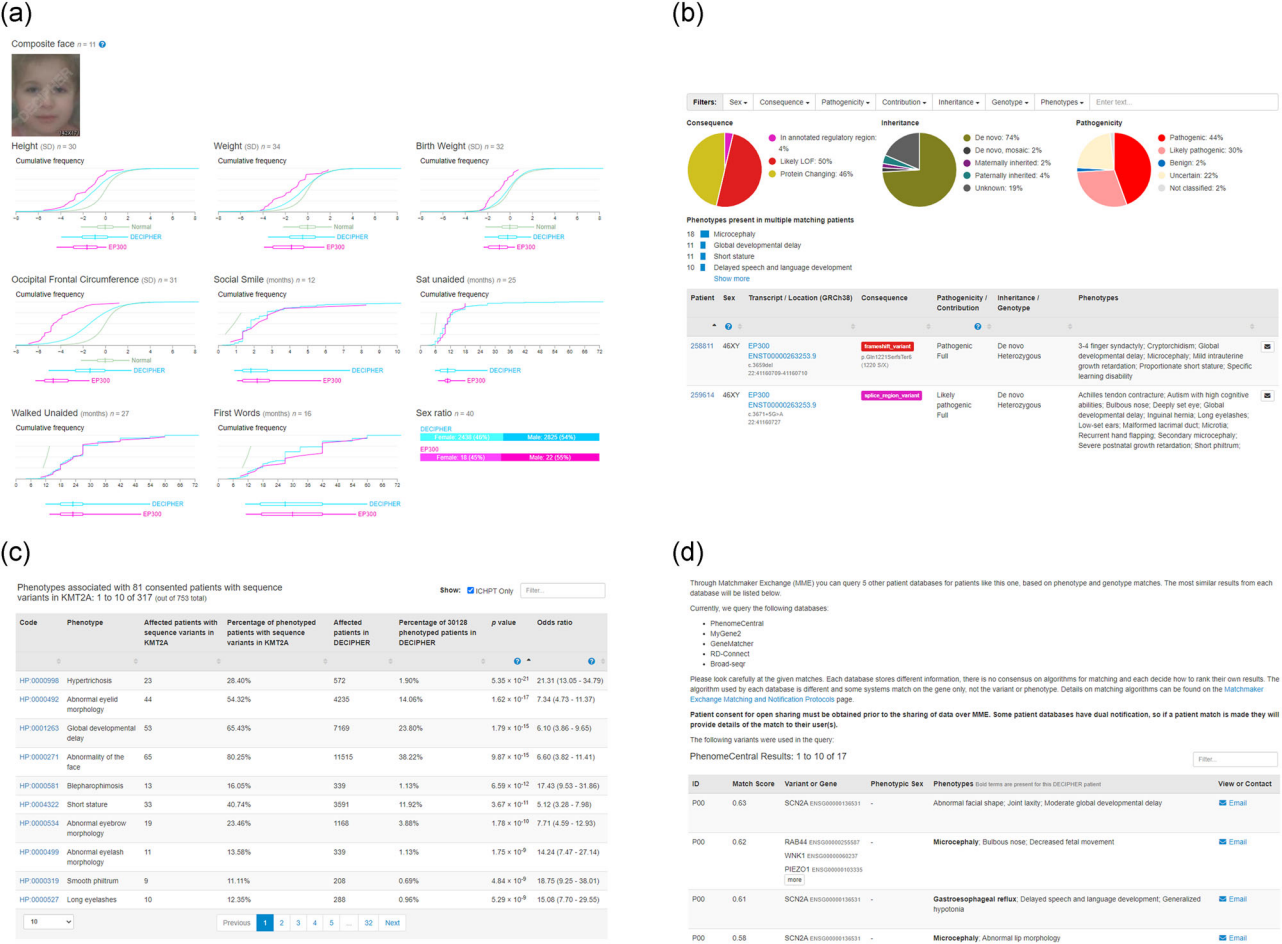
(A) Quantitative phenotype data (such as developmental milestones or anthropometric measurements) is recorded in DECIPHER and aggregated on a gene‐by‐gene basis. The data is shared openly in a series of graphs that displays expectations for the healthy population (Normal), the DECIPHER population as a whole, and the gene‐specific data. For certain genes, such as EP300 (displayed here), there are composite faces, which highlight facial dysmorphologies. (B) The matching patient interface allows users to view DECIPHER records that overlap a deposited copy‐number, sequence, or insertion variant, or a gene. In this example, the matching patients overlap EP300. Summary information is shown in a series of pi charts, along with phenotypes present in multiple matching patients. The individual patient records are displayed at the bottom of the interface. Filters are available to assist in finding the most relevant patient matches. (C) Within DECIPHER, aggregated phenotype data is used to identify the most discriminating phenotypes associated with disease genes. A table shows the percentage of phenotyped patients with sequence variants in a gene of interest, with a particular phenotype, compared with the percentage of phenotyped patients in DECIPHER with the same phenotype. The odds ratio and p value from Fisher's exact test are displayed. In this example, data for KMT2A is displayed and sorted by p value. (D): Users with write access to an open‐access patient record are able to query the MatchMaker Exchange to search for potential patient matches. DECIPHER is currently connected to Broad‐seqr, GeneMatcher, MyGene2, PhenomeCentral, and RD‐Connect. Details of potential patient matches are displayed within DECIPHER (patient IDs have been removed in this example)

Quantitative phenotype data (developmental milestones or anthropometric measurements) can be recorded in DECIPHER, and are aggregated on a gene‐by‐gene basis and shared openly (Figure 6a). In order for this information to be shown for a given gene, there must be at least five patients with both quantitative phenotype data and openly shared sequence variants annotated as pathogenic/likely pathogenic. Once this threshold is met, DECIPHER automatically aggregates and shares the information as a series of graphs on which expectations for the predominantly healthy population (Normal), the DECIPHER population as a whole, and the gene‐specific data is plotted. Anthropometric measurements are plotted around the standard deviation (adjusted for sex and gestation, where possible), while developmental milestones are plotted against time. The standard deviation for each population is displayed at the bottom of the graph as a boxplot. For users logged into DECIPHER and looking at a patient record from their center, a vertical line indicates their patient's measurement or age at attainment of the milestone, allowing them to easily judge whether it is consistent with a pathogenic/likely pathogenic variant in the gene. The display of the DECIPHER population allows users to determine if a particular measurement is particularly discriminative for a given disorder. These gene‐specific centile charts can also be used in the clinic to determine how a child is developing relative to other children with the same disorder.

#### 7.3.2 Composite facial images

For certain genes, there also are composite faces, which highlight facial dysmorphologies specific to a gene. These anonymized composite face images have been created from individuals with de novo mutations in the affected genes that were collected through the DDD study (Deciphering Developmental Disorders Study, 2017). The image capture capability in DECIPHER will facilitate further development of this aspect.

## 8 IDENTIFYING PATIENT MATCHES TO SUPPORTING DIAGNOSIS

### 8.1 Matchmaking within DECIPHER

The phenotype‐linked variant data in DECIPHER allows for effective patient matching. DECIPHER presents a powerful, flexible matching patient interface (Figure 6b), which allows users to view DECIPHER records that overlap a deposited copy‐number, sequence, or insertion variant, or a gene. The matching patient interface displays useful summary information about the potential matches, for example, for sequence variants, this is consequence, inheritance, and pathogenicity. To allow users to quickly identify the most prominent clinical features in overlapping patients, a list of phenotypes present in multiple matching patients is displayed. When viewing this interface from a patient record, additional lists showing which of the patient's phenotypes are present or not recorded in matching patients are also displayed. This allows users to easily determine if there is a good phenotypic match between their patient and other matching patients. Beneath this is a table containing information about the individual matching patient records. The table columns can be sorted and all matching phenotypes are shown in bold.

#### 8.1.1 Customizable data display

A series of filters are provided in the matching patient interface so that users can drill into the most relevant patient data. This allows users to filter on, for example, functional similarity, consequence, inheritance, and/or pathogenicity. This can be particularly useful when different variant consequences are associated with different syndromes (e.g., *SCN2A*, where loss of function variants are associated with nonspecific severe intellectual disability, and missense variants with infantile epileptic encephalopathy).

#### 8.1.2 Functionally identical variants

If the same variant has previously been deposited to DECIPHER, a “Functionally Identical Variant” interface is present, displaying variant pathogenicity and evidence, in addition to phenotype information from these patient records. This ensures that users are alerted to other patients carrying the same variant, and assists in the standardization of variant classification across centers.

#### 8.1.3 Discriminative phenotypes

The wealth of the phenotype‐genotype‐linked data in DECIPHER also allows the aggregation of data associated with pathogenic variants in disease genes. Within DECIPHER, aggregated phenotype data is used to identify the most discriminating phenotypes associated with disease genes (Figure 6c). Recognizing distinctive clinical characteristics associated with a disorder can be key to a diagnosis. The interface presents a table displaying the percentage of phenotyped patients with sequence variants in a gene of interest with a particular phenotype, compared with the percentage of phenotyped patients in DECIPHER with the same phenotype, and the odds ratio and *p *value from a Fisher's exact test, which indicate the most discriminative phenotypes associated with a gene.

#### 8.1.4 Clinician contact

If a matching patient is discovered, it is possible to contact the clinician responsible for the patient's care through DECIPHER. DECIPHER depositors are able to send messages directly, and since October 2014, over 4500 collaboration requests have been sent amongst these registered DECIPHER users. In the case where a user is not registered with DECIPHER, the DECIPHER team first moderates such contact requests, and if the request appears to be legitimate and appropriate, forwards the message to the clinician responsible for the patient, asking them to contact the requestor directly to discuss collaboration. Over 2900 such contact requests have been sent since January 2018.

### 8.2 Matchmaking through the Matchmaker Exchange

DECIPHER is a founding member of the Matchmaker Exchange (MME; https://matchmakerexchange.org), a Global Alliance for Genomics and Health (GA4GH) driver project which enables the federated discovery of similar rare disease patient data in connected databases. This worldwide collaboration allows automated matchmaking of genetic and/or phenotypic data between databases, via an application programming interface (API). Through MME, DECIPHER is currently connected to Broad‐seqr (https://seqr.broadinstitute.org/matchmaker/matchbox; Arachchi et al., 2018), GeneMatcher (https://genematcher.org; Sobreira et al., 2015), MyGene2 (https://www.mygene2.org/; MyGene2, 2016), PhenomeCentral (https://phenomecentral.org; Buske et al., 2015), and RD‐Connect (https://platform.rd-connect.eu; Lochmüller et al., 2018). Since 2020, DECIPHER depositors have made approximately 1500 requests for matches from connected databases and received details of more than 4100 potential patient matches. In the same time period, DECIPHER has received more than 55,000 requests for matches from connected databases, and has returned details of more than 255,000 potential patient matches.

Within DECIPHER, users with write access to a patient are able to query the MME. It is essential that the patient record in DECIPHER has explicit consent for open sharing, as some connected databases have dual notification, that is, they provide their user with details of any potential patient match, and unshared patient records will not be available to users of the other databases. Once MME is queried and the connected databases have responded, details of potential patient matches are displayed within the DECIPHER interface. Potential matches from each database are displayed in a tabular format with matching phenotypes in bold, assisting users in determining the level of phenotype similarity (Figure 6d). DECIPHER supports the querying of MME for patients with at least one open‐access sequence or a copy‐number variant that overlaps one gene. Other types of variants present in the patient record will not be included in the MME request.

When an MME request is sent to DECIPHER which contains genomic information, all open‐access patient sequence or copy‐number variants which overlap a single gene, and all DDD consortium research variants (see Section 8.3) are evaluated for similarity based on functional overlap. Many of the variant requests received from connected databases provide genomic coordinates in GRCh37, and in these cases, DECIPHER performs liftover to convert the coordinates to GRCh38 before identifying matches. A score for each potential patient match is provided, ranging from 0 to 1, with 1 indicating a better match. DECIPHER's scoring algorithm for genomic matches takes into account the Ensembl VEP predicted consequence, assessing the severity and similarity of the consequence to those provided in the request.

If only phenotypic data is provided, all open‐access patients with phenotypes are evaluated for a match. This takes into account all HPO ancestor terms for both the patient in the request, and patients within DECIPHER. These matches are scored by generating an Intersection over Union score comparing the HPO ancestor terms of the request patient and the patient in DECIPHER.

DECIPHER returns the 20 highest scoring matches per MME request. In the case where there are many matches, the patients' chromosomal sex is taken into account in addition to the score, to prioritize the best possible matches. The returned matches include variant, phenotype (including absent phenotypes), and diagnosis information.

## 9 DRIVING RARE DISEASE RESEARCH

**Figure 7:**
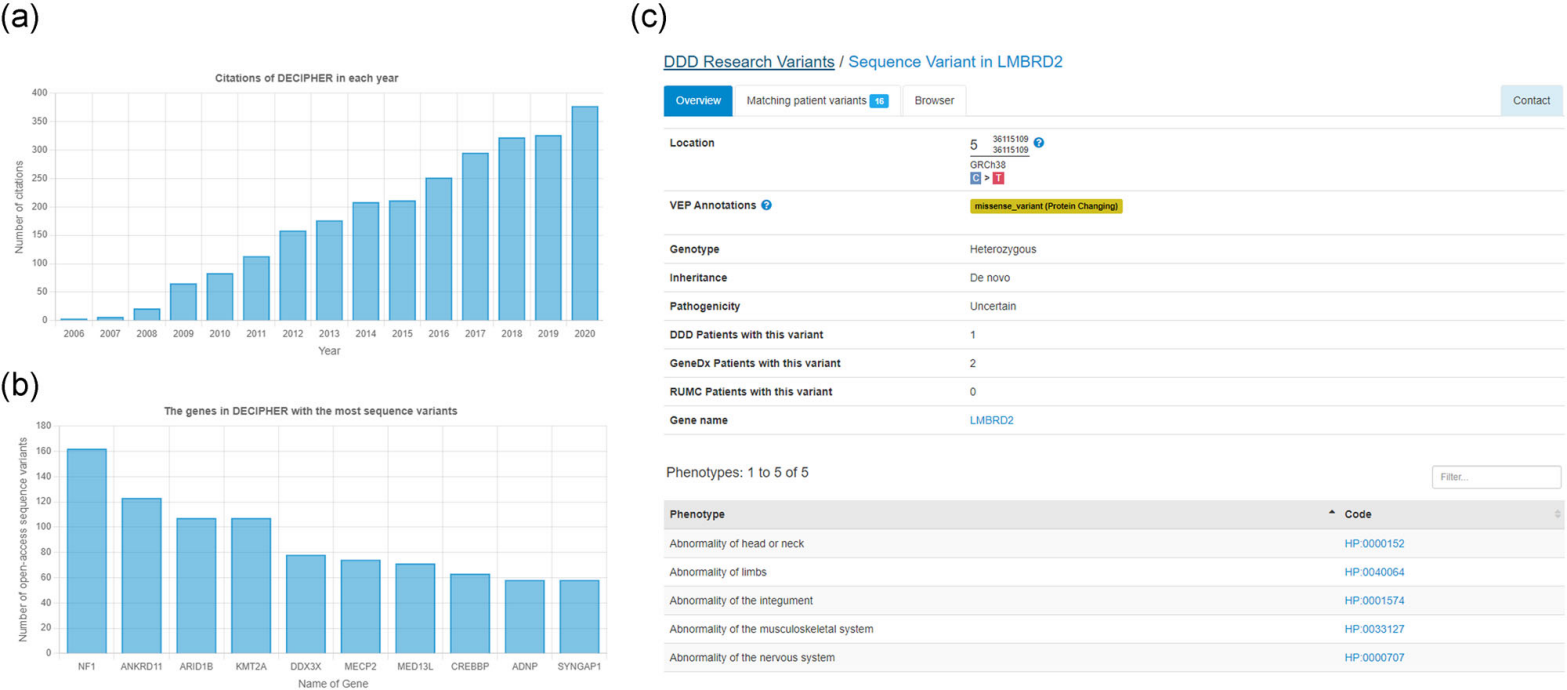
(A) Since its inception in 2004, DECIPHER has been cited in more than 2600 publications. (B) The genes with the most open‐access sequence variants in DECIPHER (at the time of writing). (C) DECIPHER openly shares variants of unknown significance identified in undiagnosed probands in the Deciphering Developmental Disorders study (research variants). For each variant, a page provides details of the variant and high‐level phenotype terms. The number of patients with each variant in the DDD data set is displayed, in addition to the number of patients identified in the GeneDx and Radboud University Medical Center de novo variant data set as described by Kaplanis et al. (2020)

The ~40,000 openly consented patient records in DECIPHER contain more than 51,000 variants and ~172,000 phenotypes, and represent a rich data set to drive rare disease research. Since its inception in 2004, DECIPHER has been cited more than 2600 times in peer‐reviewed publications (Figure 7a); a testimony to its impact on rare disease research. In some cases, there is a large genotypic patient series, which allows, for example, the full spectrum of phenotypes associated with a gene to be recognized. At the time of writing, the genes with the most open‐access sequence variants were *NF1* (162), *ANKRD11* (123), *ARID1B* (107), *KMT2A* (107), and *DDX3X* (78) (Figure 7b).

### 9.1 Search

To identify the most relevant patient records and gene information DECIPHER offers a powerful search function allowing users to search using many different categories including gene, phenotype, HPO identifier, genomic position (in GRCh37 or GRCh38), chromosome band, pathogenicity, and inheritance. Advanced searches are supported, such as searching for multiple terms either from the same category (e.g., multiple phenotypes) or different categories (e.g., gene plus phenotype). Results are displayed in a tabular format, in addition to genome browser‐based representations.

### 9.2 Driving discovery

The genotype‐linked phenotypic data allows, for example, new variant‐disease associations to be discovered, such as loss‐of‐function variants in *ARFGEF1* causing developmental delay and epilepsy (Thomas et al., 2021). The data set also enables the extension of phenotypes for new syndromes to be uncovered (e.g., Witteveen–Kolk syndrome a *SIN3A*‐related disorder; Balasubramanian et al., 2021), in addition to well‐established syndromes (e.g., *ALG13* congenital disorder of glycosylation; Alsharhan et al., 2021). It also permits the understanding of contiguous gene effects, such as that around *ERF* which causes a novel craniosynostosis syndrome with varying degrees of intellectual disability (Calpena et al., 2021).

### 9.3 DDD research variants

In addition to the openly consented patient data, DECIPHER openly shares the DDD research variants, which are variants of unknown significance identified in undiagnosed probands with developmental disorders in the DDD study. These include functional de novo variants and rare loss‐of‐function homozygous, compound heterozygous, and hemizygous variants in genes that are neither developmental disorder genes nor OMIM‐morbid genes. At present this data set comprises nearly 5000 variants. High‐level phenotype terms are provided for each variant (Figure 7c). The number of patients with each variant in the DDD data set is displayed, in addition to the number of patients identified in the GeneDx and Radboud University Medical Center de novo variant data set as described by Kaplanis et al. (2020). This data set enables the discovery of new gene‐disease associations.

### 9.4 Bulk data for research

The openly consented patient data is available for bulk download for research purposes, subject to a data access agreement. In bulk, the data can be used, for example, for developing new analytical methods, in understanding patterns of polymorphism, and in refining critical intervals to map genes involved in specific phenotypes and diseases. The data set has recently been used to associate phenotypes with functional systems (Jabato et al., 2021), and to develop a new tool to assist clinical interpretation of CNVs (Requena et al., 2021). DECIPHER also shares the data in bulk for display, subject to a Data Display Agreement. This allows third‐party variant analysis companies and academic genome browser providers such as Ensembl and UCSC to display the data, maximizing the possibility of finding patient matches.

## 10 Summary and future plans

DECIPHER is a free web‐based platform that enables the visualization of genomic and phenotypic relationships to aid variant interpretation, diagnosis, and discovery. The platform supports the interpretation and sharing of almost all types of genetic variation, providing variant interpretation interfaces that contextualize the genotypic and phenotypic data. These interfaces include a genome browser, protein browser, matching patient variant displays, and tools to assess the variant according to internationally‐accepted standards. Potential matching patients in other connected databases can also be identified through the MME. The platform enables the flexible and proportionate sharing of patient‐level data, so that the depth and breadth of sharing is tailored to the scientific/clinical needs and the level of patient consent attained. DECIPHER currently openly shares ~40,000 rare disease patient records, and supports the more limited sharing of >63,000. DECIPHER is under continuous development, ensuring that it keeps up to date with the fast‐moving field of rare genetic diseases. New user‐facing features are released approximately every 6 weeks, along with updates to reference data sources (such as the Ensembl/GENCODE gene set, HPO, ClinVar). Future plans for the platform include integration of datasets to further assist variant interpretation in the noncoding genome (e.g., regulatory datasets), inclusion of management resource information (e.g., treatment information and links to cellular pathway information), and integration of functional data (e.g., saturation genome editing).

DECIPHER enables clinical use of selected new datasets and tools developed by the research community. This makes them directly available to clinicians and clinical scientists, thereby assisting in the rapid translation of research into the diagnostic arena. Since its inception in 2004, the platform has made a huge impact on rare genetic disease research and is cited in more than 2600 publications. The rich phenotype‐linked variant data hosted by DECIPHER, and the tools it provides, enable DECIPHER to advance its mission of mapping the clinically relevant parts of the genome.

## CONFLICT OF INTERESTS

Matthew Hurles is a cofounder, shareholder, and nonexecutive director of Congenica Ltd., a diagnostic software company.
